# Assessment of Micafungin Dosage Regimens in Patients with Cancer Using Pharmacokinetic/Pharmacodynamic Modeling and Monte Carlo Simulation

**DOI:** 10.3390/antibiotics10111363

**Published:** 2021-11-08

**Authors:** Saeed Alqahtani, Asma Alfarhan, Abdullah Alsultan, Emad Alsarhani, Abdulaziz Alsubaie, Yousif Asiri

**Affiliations:** 1 Department of Clinical Pharmacy, College of Pharmacy, King Saud University, Riyadh 11451, Saudi Arabia; asmalfarhan@KSU.EDU.SA (A.A.); absultan@ksu.edu.sa (A.A.); ealsarhani@KSU.EDU.SA (E.A.); abdalsbaie@ksu.edu.sa (A.A.); yasiri@ksu.edu.sa (Y.A.); 2 Clinical Pharmacokinetics and Pharmacodynamics Unit, King Saud University Medical City, Riyadh 11451, Saudi Arabia

**Keywords:** micafungin, cancer, population pharmacokinetics, modeling, Monte Carlo simulation

## Abstract

Micafungin is widely used for invasive candidiasis, especially in critically ill patients and those with cancer, and for empirical antifungal therapy in patients with neutropenic fever. This is the first study to investigate the pharmacokinetics and disposition parameters of micafungin in patients with cancer. In this observational pharmacokinetic study, blood samples were collected and analyzed using high-performance liquid chromatography. Pharmacokinetic parameters were estimated using Monolix 4.4 software. The plasma micafungin concentrations were measured in a total of 133 samples from 19 patients. In the final two-compartment model with linear elimination, the estimated micafungin clearance (CL) was significantly higher in patients with cancer than in those without cancer (1.2 vs. 0.6 L/h, *p* = 0.012), whereas other parameters did not significantly differ between the two groups. Aspartate and alanine transaminases and body weight significantly influenced micafungin CL in patients, with and without cancer. Overall, the probability of target attainment increased with increasing doses and decreased with higher MICs in both groups. In simulations, the patients without cancer achieved higher pharmacokinetic/pharmacodynamic targets with a 90% probability for all simulated doses, compared to the patients with cancer. Micafungin demonstrated dose-proportional linear pharmacokinetics in both the patients with and those without cancer. The estimated micafungin CL was significantly higher in patients with cancer, suggesting a need for increased dosage, especially for *Candida* spp. with high MICs, in these patients. Further studies should assess the efficacy and optimum dosage of micafungin for the treatment and prevention of febrile neutropenia (FN) in patients with cancer.

## 1. Introduction

Neutropenia is a common, serious complication of myelosuppressive chemotherapy and a leading cause of infection-related morbidity and mortality in patients with cancer [1]. Febrile neutropenia (FN) is a serious condition that continues to have major clinical, economic, and quality-of-life impacts on chemotherapy-treated patients [2,3,4]. The incidence of FN is lower (5–10%) in patients with solid tumors receiving cytotoxic therapy, who are at low risk from medical complications, compared to the higher FN incidence rates of 20–25% and 85–95% in those with non-leukemic hematologic malignancies and acute leukemia, respectively [5,6]. Furthermore, FN mortality ranges from 8% to 14% [7,8], and bacterial infections are the most common cause of FN [9,10]. Fungal infections are prevalent in high-risk patients with neutropenic fever, and patients with prolonged and severe neutropenia are at higher risk of candidemia and invasive fungal infections. *Candida albicans* is the most prevalent yeast pathogen responsible for fungal infections in neutropenic patients with cancer. Other *Candida* spp., including *C. glabrata* and *C. tropicalis*, are also prevalent, whereas *Aspergillus* spp. are the most common pathogenic molds in hematological malignancies [11,12].

Effective strategies to anticipate, prevent, and treat this infection have resulted in better outcomes. Recently released clinical practice guidelines recommend the use of echinocandins, including anidulafungin, micafungin, and caspofungin, as first-line antifungals for the primary and secondary prevention of FN in high-risk patients [13,14,15]. These antifungals are also recommended as empirical antifungal therapy in patients with persistent FN [13,14,15]. Compared to other systemic antifungals, the advantages of echinocandins include a relatively better toxicity profile and less potential for drug-drug interactions [16].

Micafungin shows excellent antifungal activity against the majority of *Candida* spp., including azole-resistant strains, and it shows some activity against *Aspergillus* spp. [17]. Although all echinocandins share a similar spectrum of activity, each agent differs in its pathway of metabolism, resulting in distinguishable half-lives, dosing strategies, and drug interaction profiles [18]. Echinocandins are minimally absorbed after oral administration due to their large molecular weights and are, therefore, available only as intravenous formulations [17]. All three echinocandins are highly bound to plasma proteins (85–99%) [17] and have relatively low volumes of distribution [17]. Moreover, echinocandins distribute minimally to urine, the eyes, and the cerebrospinal fluid [17]. In addition, echinocandins are not significantly metabolized by cytochrome P450, nor are they inhibitors or substrates of P-glycoprotein drug efflux pumps. Thus, echinocandins display a lower potential for drug-drug interactions, which reduces their likelihood to be targets of drug-drug interactions, compared to other antifungals [17].

Echinocandins exhibit concentration-dependent killing against *Candida* spp., based on in vivo studies showing that echinocandins have a fungicidal effect proportional to the maximum (peak) plasma drug concentration, and a persistent antifungal effect after the reduction in plasma drug concentration to levels below the MIC [19,20]. Fungicidal efficacy against *Candida* spp. is also correlated with the 24 h area under the concentration-time curve (AUC_0–24_)/MIC ratio [19,20].

Physiological changes in patients with cancer may result in altered pharmacokinetic parameters and may lead to either supratherapeutic or subtherapeutic levels of antimicrobial agents, thereby affecting their efficacy and safety [21]. Although the pharmacokinetics of micafungin have been investigated in various patient populations, including those in surgical, intensive care unit, and lung transplantation settings, no study to date has evaluated the pharmacokinetic properties of micafungin in adult patients with cancer [22,23,24,25]. Therefore, in this study, we compared the pharmacokinetics of micafungin between patients with and without cancer and developed a model to describe the complex pharmacokinetics of micafungin in both patient groups. We investigated inter-individual variability and assessed the probability of achieving PK/PD targets with current dosing strategies in this patient population, with the overarching aim of providing the basis for establishing an effective and safe micafungin dose regimen for patients with cancer.

## 2. Methods and Patients

### 2.1. Study Design and Subjects 

This was a prospective pharmacokinetic study to compare adult patients with and without cancer. The study was conducted at King Saud University Medical City, a tertiary hospital. The study included male and female patients aged ≥ 18 years, who were admitted to the hospital and received a minimum of 2 micafungin doses based on empirical evidence or culture results. Patients who were pregnant and those with a micafungin allergy were excluded. The study protocol was approved by the Institutional Review Board of King Saud University Medical City (E-18-3373), and written informed consent was obtained from all patients before their inclusion in the study.

### 2.2. Micafungin Dosing and Blood Sampling

All patients were treated with a 100–150 mg daily dose of micafungin for empirical use or confirmed fungal infection. Micafungin was diluted in 100 mL isotonic saline solution and infused over 60 min. Blood samples were drawn at 1, 2, 4, 6, 8, 12, and 24 h from the initiation of the micafungin dose. All blood samples were centrifuged, and plasma samples were stored at −80 °C until analysis.

### 2.3. Analytical Assay

Plasma micafungin concentrations were determined using a high-performance liquid chromatography (HPLC) method with a UV detection system, with slight modification [26]. Plasma samples were separated from whole blood, acidified with phosphoric acid, precipitated with acetonitrile, and centrifuged prior to dilution with buffer and injection into an HPLC system. Anidulafungin served as I.S. for micafungin. The method was fully validated and achieved using an isocratic Prominence Shimadzu HPLC system (Shimadzu, Columbia, MD, USA), which comprised an autosampler (SIL-20AHT), a UV detector (Shimadzu UV SPD-20A), and a pump (LC-20AB) connected to a degasser (Dgu-20A3). The separation of micafungin was performed on a Phenomenex Luna C18 column (250 × 4.6 mm i.d., 5 µm; Phenomenex, CA). The mobile phase comprised 0.1% (*w*/*w*) ammonium acetate in water, with pH adjusted to 7.0 with 25% NH_3_ solution and acetonitrile (70%:30%); the flow rate was 1 mL/min, and micafungin was detected at 273 nm. Data acquisition was achieved using LC Solution chromatographic software version 1.22 SP1 (Shimadzu, Columbia, MD, USA). 

### 2.4. Population Pharmacokinetic Modeling

Data from patients with and without cancer were comodeled using Monolix 4.4 software (Supplementary File S1). Monolix estimates pharmacokinetic parameters using the stochastic approximation expectation maximization algorithm [27]. In the first step, we developed the base structural model for micafungin by fitting various compartmental models to plasma drug concentrations. The following criteria were followed to select between models: (a) a decrease in the minimum of the objective function value (log-likelihood value); (b) precision of the parameter estimation expressed as RSE (%) and calculated as the ratio between the standard error and final parameter estimate; (c) physiologic plausibility; and (d) GOF plots including observed and predicted concentrations, residuals plot, and visual predictive check [28,29].

### 2.5. Covariate Model

A covariate model was developed in a stepwise fashion, with forward inclusion based on model selection criteria. First, we plotted the empirical Bayesian estimates vs. covariates to screen for potentially significant correlations. Next, we performed a stepwise regression analysis to test the significant covariates identified in step 1, using the −2 log-likelihood ratio test [28,29]. Age, body weight, height, BMI, sex, serum creatinine, creatinine clearance, sequential organ failure assessment score, and levels of AST, alanine transaminase, total bilirubin, and albumin were tested as covariates.

### 2.6. Model Diagnostics

GOF was assessed using the log-likelihood criterion during model building. Plots of model-based individual and population predictions vs. the observed concentrations were performed for model assessment. A predicted–corrected visual predictive check using 1000 simulations was constructed to study the performance of the final model. A statistically significant improvement in log-likelihood value (*p* < 0.05) was required for a more complex model to be supported.

### 2.7. Monte Carlo Simulations of Plasma Concentrations and PTA

Various Monte Carlo simulations (n = 1000) of plasma concentrations were employed using R statistical software to calculate the AUC_0–24_/MIC for varying MICs (0.002–4 mg/L). The final population model was utilized to simulate different micafungin dosing regimens. Intravenous doses of 100, 150, and 200 mg/day micafungin were simulated for patients weighing 50, 70, and 100 kg with AST and ALT levels of 2–3 × ULM. MIC data for *Candida* spp. and *Candida parapsilosis* were extracted from the European Committee on Antimicrobial Susceptibility Testing database [30] and used to determine PTA. Target micafungin pharmacokinetic/pharmacodynamic values were defined according to total plasma AUC_0–24_/MIC targets of 3000 and 285 for non-*parapsilosis Candida* spp. and *Candida parapsilosis*, respectively [31]. *A priori*, a dosing regimen was considered successful if the fractional target attainment was >90%.

## 3. Results

### 3.1. Study Cohort

The cohort of this prospective, single-center study comprised 19 patients, including 10 patients with cancer and 9 without cancer. As shown in Table 1, there were no differences in the demographic and clinical characteristics between the two groups. All patients with cancer received 100 mg/day micafungin, while seven patients without cancer received 100 mg/day micafungin, and two patients without cancer received 150 mg/day.

### 3.2. Population Pharmacokinetic Modeling

The plasma micafungin concentrations were measured in seven samples collected from each of the 19 patients, for a total of 133 samples. The final model was a two-compartment model with linear elimination from the central compartment. A combined error model was the most accurate for residual and interpatient variability. The pharmacokinetic model was parameterized in terms of clearance (CL), volume of the central compartment (V1), volume of the peripheral compartment (V2), and intercompartmental clearance (Q). Table 2 shows the parameter estimates of the model for both groups. The estimated CL was significantly higher in patients with cancer than in those without cancer (1.2 vs. 0.6 L/h, *p* = 0.012), while there were no differences in other parameters between the two groups. Among the investigated covariates, aspartate transaminase (AST), alanine transaminase (ALT), and body weight significantly influenced the CL of micafungin in both patient groups. On the other hand, body mass index (BMI), body weight, and levels of total bilirubin and albumin affected the V1 in both groups.

**Figure 1:**
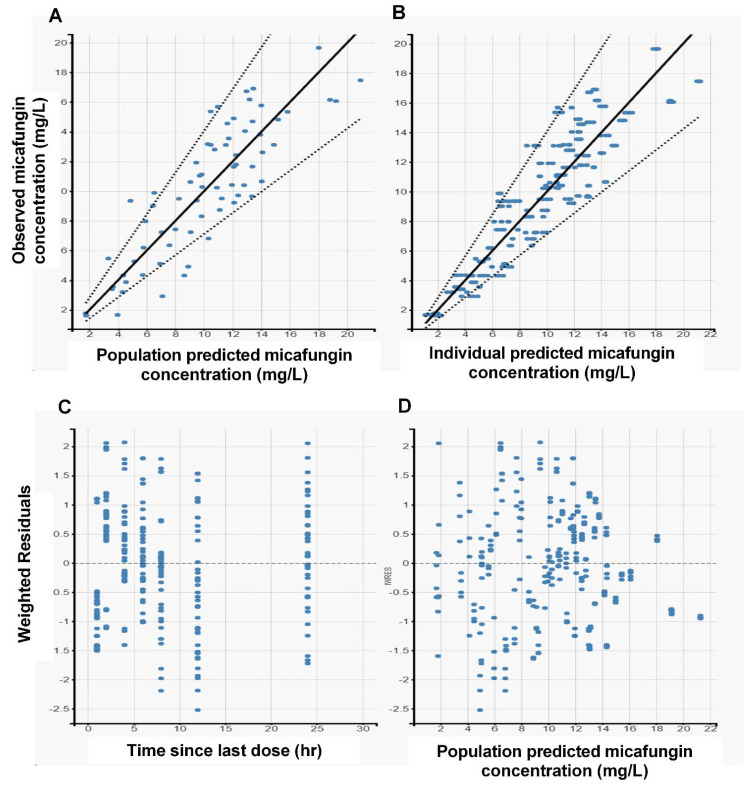
Goodness−of−fit (GOF) plots obtained from the final model for micafungin. (**A**) Individual predictions of micafungin vs. observed concentrations. (**B**) Population predictions of micafungin vs. observed concentrations. (**C**) Weighted residuals vs. time since last dose. (**D**) Weighted residuals vs. population predicted concentrations.

**Figure 2:**
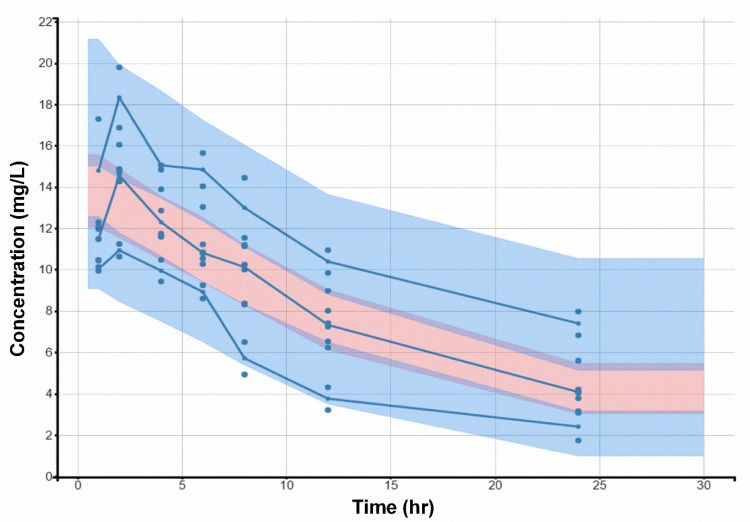
Predicted–corrected visual predictive check (pcVPC) (shaded areas) and observed data (circles) for micafungin concentration vs. time based on 1000 Monte Carlo simulations. Solid blue lines represent the 10th, 50th, and 90th percentiles of the observed data. Shaded regions represent 90% confidence interval around the 10th (Below blue shaded region), 50th (Middle pink shaded region), and 90th (Above blue shaded region) percentiles of the simulated data.

The diagnostic goodness-of-fit (GOF) plots for the final micafungin covariate model (Figure 1) did not show major deviations. As shown in Table 2, the relative standard errors (RSEs [%]), revealed that all parameters were precisely estimated. The predicted–corrected visual predictive check of the final model (Figure 2) revealed that the predictions were consistent with the observations, suggesting good validity of the model to the data. As shown in Figure 1 and Figure 2, the final pharmacokinetic model adequately described the measured concentrations. As a result, the model was used to simulate all subsequent dosing regimens.

### 3.3. Monte Carlo Simulations of Plasma Concentrations and Probability of Target Attainment

**Figure 3:**
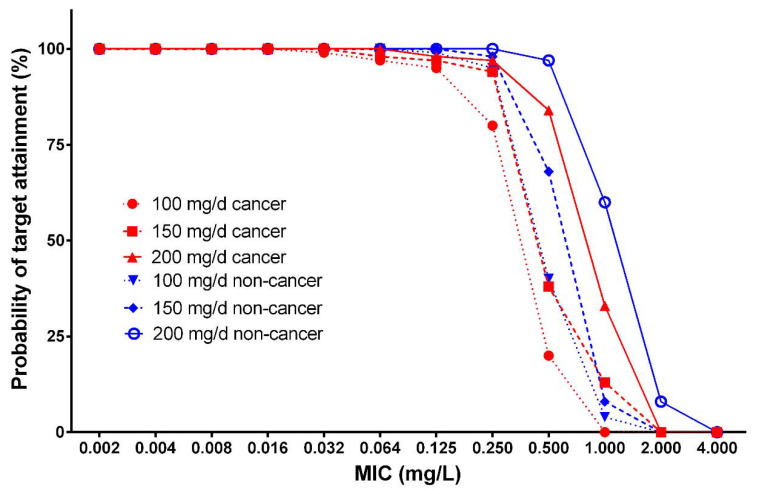
Probability of target attainment, using a 24 h area under the concentration-time curve (AUC_0–24_)/MIC target of 285 for *Candida parapsilosis* for different micafungin dosing regimens in patients with and without cancer.

**Figure 4:**
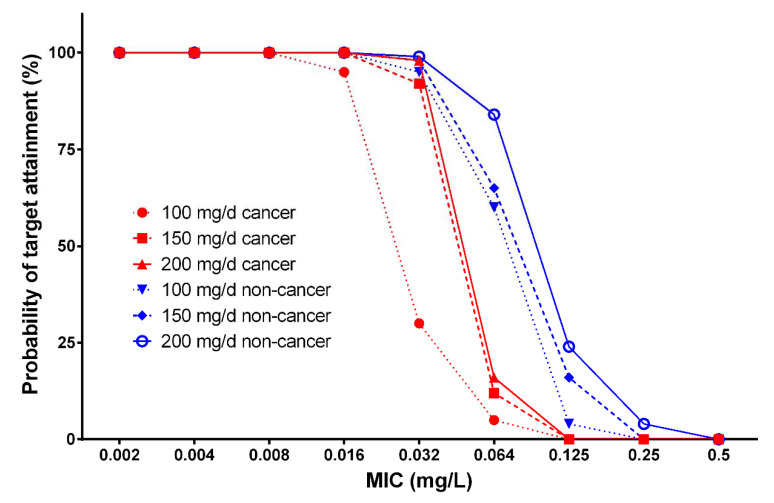
Probability of target attainment, using an AUC_0–24_/MIC target of 3000 for non-*parapsilosis Candida* spp. for different micafungin dosing regimens in patients with and without cancer.

We used Monte Carlo simulations to evaluate different micafungin dosing regimens. The probability of target attainment (PTA) values for different micafungin dosing regimens against the MIC distributions for *Candida* spp. and *C. parapsilosis* are presented in Figure 3 and Figure 4. Overall, the PTA increased with increasing doses and decreased with higher MICs for both patient groups. In the simulations, the patients without cancer achieved higher pharmacokinetic/pharmacodynamic (PK/PD) targets, with 90% probability for *Candida* spp. and *C. parapsilosis*, and higher MICs for all simulated doses, when compared with the patients with cancer. In addition, the simulation of changes in body weight revealed differences in PTA values for *Candida* spp. and *C. parapsilosis* in both groups (Table 3). On the other hand, the simulation of changes in ALT and AST levels did not affect the PTA values.

## 4. Discussion

FN poses a serious risk and causes high rates of treatment failure in patients with cancer. The FN-associated mortality is 8–15% in patients with cancer [7,8]. In addition, FN is a common cause of delays in chemotherapy schedules and dose reduction, which have a negative impact on final treatment outcomes [2]. Furthermore, FN is a major cause of prolonged hospitalization and has substantial impact on patients’ quality of life [3,4]. Bacterial infections are the most common cause of FN [9,10]; however, fungal infections are prevalent in high-risk patients. *C. albicans* is the predominant yeast pathogen isolated in patients with cancer, whereas other *Candida* spp., including *C. glabrata*, *C. tropicalis*, and *C. parapsilosis*, are also prevalent in these patients [11,12]. Thus, the early recognition of patients at high-risk for FN and the appropriate initiation of prophylactic antibacterial and/or antifungal agents are of utmost importance [14]. According to the recently published guidelines for the management of patients with cancer diagnosed with FN, echinocandins, including micafungin and caspofungin, are recommended as first-line therapeutic options for the primary and secondary prevention of fungal infections in high-risk patients. Echinocandins are also recommended as the first-line empirical antifungal therapy in patients with persistent FN [13,14,15].

Micafungin follows linear elimination pharmacokinetics, producing a terminal half-life of approximately 15 h in adults [19]. Micafungin is metabolized in the liver by catechol O-methyltransferase, arylsulfatase, and hydroxylation [17]. Only 1% of the unchanged drug is excreted through the kidneys [17]. Micafungin is non-dialyzable and does not require dose adjustment in patients with renal impairment [17]. The elimination pharmacokinetics of micafungin in patients with advanced hepatic insufficiency are not fully understood [20]. Micafungin exhibits concentration-dependent killing of *Candida* spp., and the AUC_0–24_/MIC is the best pharmacodynamic parameter for describing the dose-response relationship [19,20].

Physiological changes in patients with cancer may result in altered pharmacokinetic parameters [21]. Several changes in distribution and metabolism processes lead to these observed changes in the PK parameters [21]. Changes in the plasma proteins (including albumin) have a direct impact on the volume of distribution and the quantity of drugs available for metabolism [21]. As shown in previous pharmacokinetic studies on patients with cancer, these changes can lead to either supratherapeutic or subtherapeutic levels of antimicrobial agents [32,33,34], subsequently affecting their efficacy and safety. No study to date has investigated the pharmacokinetics of micafungin in patients with cancer [22,23,24,25].

In this prospective study, we investigated the pharmacokinetics of micafungin in patients with cancer compared to those without cancer. We described the plasma concentration-time curve of micafungin using a two-compartment model, similar to that described in previous studies [22,23,24,25,32,33,34,35,36,37,38,39]. The estimated CL, V1, Q, and V2 values for micafungin were 1.2 L/h, 10.7 L, 0.144 L/h, and 3.5 L, respectively, in patients with cancer and 0.6 L/h, 12 L, 0.188 L/h, and 2.77 L, respectively, in patients without cancer. Overall, there were no differences in the pharmacokinetic parameters except for the estimated micafungin CL between the two groups. The significantly higher estimated CL found in patients with cancer, compared to those without cancer, might be attributable to increased hepatic blood flow due to the modulation of vascular activity of liver tissue [40,41]. Other possible reasons for such differences include the increased free fraction of micafungin due to changes in plasma proteins levels or other, yet unknown, reasons in patients with cancer, which warrant further investigation [40,41]. Strikingly, the micafungin CL in both groups was significantly lower than that reported in other populations (0.84 L/h) [22,23,24,25,32,33,34,35,36,37,38,39]. Among the investigated covariates, body weight significantly influenced the micafungin CL in both groups, a finding that is in line with other pharmacokinetic studies, suggesting that obese patients may need higher doses of micafungin [40,41]. ALT and AST were the other parameters with a significant influence on CL, an expected finding given that micafungin is primarily eliminated via hepatic metabolism [20]. Conversely, BMI, body weight, total bilirubin, and albumin affected the V1 in both groups, which accords with previous studies [22,23,24,25,32,33,34,35,36,37,38,39]. Although several studies reported a correlation between disease severity and micafungin CL, we were not able to identify the sequential organ failure assessment score as a relevant covariate [23,24].

All micafungin dosing regimens evaluated using Monto Carlo simulations were adequate in obtaining optimal PTAs of ≥90% for both *C. parapsilosis* and *non-Candida parapsilosis* spp., with MICs of ≤0.016 mg/L in both the patients with cancer and those without cancer. For *C. parapsilosis*, all dosage regimens in both groups provided PTAs of ≥90% for MICs of ≤0.250 mg/L, except for the dosing regimen of 100 mg/day micafungin in patients with cancer, which was able to achieve the PK/PD target only at an MIC of ≤0.125 mg/L. The simulated dosage regimen of 200 mg/day micafungin was the only optimal regimen to achieve PTAs of ≥90% at an MIC of 0.500 mg/L in patients without cancer. Unfortunately, in patients with cancer, none of the evaluated regimens were optimal at an MIC of ≥0.500 mg/L. At an MIC of ≥1.000 mg/L, none of the simulated dosing regimens were sufficient to achieve good target attainment in either group. For non-*parapsilosis Candida* spp., 100 mg/day micafungin was sufficient to achieve a 90% PTA at MICs of ≤0.032 mg/L in patients without cancer, whereas the same dosage regimen failed to achieve the PK/PD target at an MIC of 0.032 mg/L (approximate PTA of 30%) in patients with cancer. PTA exceeded 90% at an MIC of 0.032 mg/L with the dosing regimen of 150 mg/day micafungin in both the cancer and non-cancer groups, whereas the same dosing regimen was insufficient to achieve a 90% PTA at MICs of ≥0.064 mg/L in both groups. For an MIC of 0.064 mg/L, only the dosage regimen of 200 mg/day micafungin provided a PTA exceeding 90% in patients without cancer, whereas all regimens were associated with suboptimal PTA for MICs of ≥0.064 mg/L in patients with cancer. In short, PTA increased with increasing doses and decreased with higher MICs for both groups. In the simulations, the patients without cancer achieved higher PK/PD targets with a 90% probability for *Candida* spp. and *C. parapsilosis* with higher MICs for all evaluated doses, compared to the patients with cancer. Moreover, the simulations including changes in body weight revealed differences in PTA for *Candida* spp. and *C. parapsilosis* in both groups (Table 3). In contrast, the changes in ALT and AST levels did not affect the PTA in the simulations.

To our knowledge, this is the first study comparing the pharmacokinetics of micafungin between patients with and without cancer. Nonetheless, the study findings should be interpreted with consideration given to several limitations. First, the relatively small cohort of patients might have prevented the identification of other significant covariates that could predict variability in pharmacokinetic parameters. Second, in the present study we measured total micafungin concentrations (unbound plus bound) and not the unbound or free fractions. Third, most of the study patients were terminally ill and on multiple medications, with different health statuses and disease severity, which prevented the assessment of safety and clinical outcomes. Finally, the study comprised a heterogeneous group of patients with various cancers in various stages, which might have led to significant interpatient variability.

## 5. Conclusions

Micafungin demonstrated dose-proportional linear pharmacokinetics in patients with cancer, consistent with that observed in patients without cancer. The estimated micafungin CL was significantly higher in patients with cancer, suggesting a need for increased dosage in this patient population, especially for *Candida* spp. with high MICs. Nonetheless, further studies are warranted to assess the efficacy and optimum dosage of micafungin for the treatment and prevention of FN in patients with cancer.

## Figures and Tables

**Table 1:** Clinical and demographic characteristics of patients included in the study.

Characteristics	Patients with Cancer (n = 10)	Patients without Cancer (n = 9)	*p* Value
**Age, years, mean (SD)**	47.3 (12.3)	51.1 (19.1)	0.25
**Sex, %** **male/female**	60/40	67/33	0.35
**Weight, kg, mean (SD)**	63.4 (18.2)	69.8 (15.7)	0.23
**Height, cm, mean (SD)**	162.2 (9.9)	163.1 (7.3)	0.78
**Serum creatinine, mmol/L, mean (SD)**	74.7 (43.4)	63.6 (35.8)	0.16
**CL_Cr_, mL/min, mean (SD)**	103 (58.8)	99 (69.3)	0.97
**Albumin, mean (SD)**	25.6 (5.8)	22.6 (3.7)	0.12
**AST, mean (SD)**	34.2 (9.3)	37.7 (15.4)	0.14
**ALT, mean (SD)**	26.3 (11.3)	28.3 (6.7)	0.23
**Total bilirubin, mean (SD)**	26.5 (3.8)	20.5 (16.4)	0.31
**SOFA score**	7 (5.5)	8 (6.5)	0.25

AST, aspartate transaminase; ALT, alanine transaminase; CL_Cr_, creatinine clearance; SOFA, sequential organ failure assessment.

**Table 2:** Population pharmacokinetic parameter estimates of micafungin following intravenous infusion.

Parameter	Patients with Cancer		Patients without Cancer		
	Estimate	RSE (%)	Estimate	RSE (%)	*p* Values
CL (L/h)	1.2	11.6	0.6	14	0.012
V1 (L)	10.7	23.6	12	22.2	0.65
Q (L/h)	0.144	14	0.188	10	0.56
V2 (L)	3.5	16	2.77	12.5	0.73
IIV ** for CL (%)	34.1	14.8	11.8	18	
IIV for V1 (%)	7.6	5.2	7.6	20	
IIV for Q (%)	32.2	18	20.4	13	
IIV for V2 (%)	36.8	15	32.1	22	
Residual error					
a	0.21	10.7	0.15	9.2	
b	0.22	4.5	0.18	13.6	

** Expressed as coefficient of variation. CL, clearance; IIV, inter-individual variability; Q, intercompartmental clearance; RSE, relative standard error; V1, volume of the central compartment; V2, volume of the peripheral compartment.

**Table 3:** MIC breakpoints for *Candida* spp. and *C. parapsilosis* indicating 90% PTA for various micafungin doses in patients weighing 50, 70, and 100 kg.

Micafungin Dose (mg)	Body Weight (kg)	MIC Breakpoint (mg/L)			
		Patients with Cancer		Patients without Cancer	
		*Candida* spp.	*C. parapsilosis*	*Candida* spp.	*C. parapsilosis*
100	50	0.032	0.25	0.032	0.5
	70	0.016	0.125	0.032	0.25
	100	0.016	0.064	0.016	0.125
150	50	0.032	0.25	0.064	0.5
	70	0.032	0.25	0.032	0.25
	100	0.016	0.125	0.032	0.25
200	50	0.032	0.25	0.064	0.5
	70	0.032	0.25	0.032	0.5
	100	0.016	0.125	0.032	0.5

PTA, probability of target attainment.

## Supplementary Materials

The following are available online at https://www.mdpi.com/article/10.3390/antibiotics10111363/s1, File S1: Methods.
