# Supplementary material for: Assessment of Micafungin Dosage Regimens in Patients with Cancer Using Pharmacokinetic/Pharmacodynamic Modeling and Monte Carlo Simulation
Source: Antibiotics (Basel). 2021 Nov 8;10(11):1363. doi: 10.3390/antibiotics10111363 (PMC8615109; doi:10.3390/antibiotics10111363)
Supplement: Supplementary file 1 [file antibiotics-10-01363-s001.zip › File S1.pdf]

antibiotics

MDPI

Assessment of Micafungin Dosage Regimens in Patients with Cancer Using Pharmacokinetic/Pharmacodynamic Modeling and Monte Carlo Simulation

Methods

Population Pharmacokinetic Modeling

Data from patients with and without cancer were comodeled using the Monolix 4.4 software. Monolix estimates pharmacokinetic parameters using the stochastic approximation expectation maximization algorithm (27).

The nonlinear mixed effects model was defined as

$$y_{ij} = f(x_{ij}; \phi_i) + g(x_{ij}; \phi_i)\varepsilon_{ij} \quad ; \quad \varepsilon_{ij} \sim N(0, \sigma^2)$$

where f is the parametric function of the structural model, g is the parametric function for the error model, y_{ij} is the jth observation in the ith subject, φ_1 is the p-dimensional vector of model parameters for the ith subject, x_{ij} are the design variables for the jth observation in the ith subject, ε_{ij} is the residual error for the jth observation, in the ith subject and σ^2 is the variance of the residual unidentified variability. In most cases $g=f$, i.e. a proportional error model, or $g=1$, i.e. an additive error model.

The random effect was defined as

A log-normal random effect was assumed for intersubject variability on each structural PK parameter and combined error model was assumed for the residual-error model.

$$CL_j = CL_{pop} \times \exp(\eta_j) \text{ and } C_{obs,ij} = C_{pred,ij} \times (1 + \varepsilon_{prop,ij}) + \varepsilon_{const,ij},$$

where CL is the structural parameter for the jth individual, CL_{pop} is the typical value for the parameter CL in the population, and η_j is a random variable representing intersubject variability of the parameter, with mean zero and variance ω^2. The variables $C_{obs,ij}$ and $C_{pred,ij}$ represent the ith observed and predicted concentrations, respectively, for the jth patient, while $\varepsilon_{prop,ij}$ and $\varepsilon_{const,ij}$ are the proportional or constant random residual errors, which are normally distributed with mean zero and variance σ_{prop}^2 or σ_{const}^2

Covariate Model

A covariate model was developed in a stepwise fashion with forward inclusion based on model selection criteria. First, we plotted the empirical Bayesian estimates vs. covariates to screen for potentially significant correlations. Next, we performed a stepwise regression analysis to test the significant covariates identified in step 1 using the −2 log-likelihood ratio test.

The random effect and covariate model was defined as

$CL_j = CL_{pop} \times \exp(\eta_j)$ and $C_{obs,ij} = C_{pred,ij} \times (1 + \varepsilon_{prop,ij}) + \varepsilon_{const,ij}$,

where CL is the structural parameter for the *j*th individual, CL_{pop} is the typical value for the parameter CL in the population, and η_j is a random variable representing intersubject variability of the parameter, with mean zero and variance ω^2. The variables $C_{obs,ij}$ and $C_{pred,ij}$ represent the *i*th observed and predicted concentrations, respectively, for the *j*th patient, while $\varepsilon_{prop,ij}$ and $\varepsilon_{const,ij}$ are the proportional or constant random residual errors, which are normally distributed with mean zero and variance σ_{prop}^2 or σ_{const}^2
